# Rapid Synthesis of Robust Antibacterial and Biodegradable Hydrogels via Frontal Polymerization

**DOI:** 10.3390/gels9120920

**Published:** 2023-11-21

**Authors:** Jinze Wang, Hao Li, Hai-Xia Shen, Wei Zhao, Qing Li, Cai-Feng Wang, Su Chen

**Affiliations:** State Key Laboratory of Materials-Oriented Chemical Engineering, College of Chemical Engineering, Nanjing Tech University, No. 5 Xin Mofan Road, Nanjing 210009, Chinaleo541437118@163.com (H.L.); shx@njtech.edu.cn (H.-X.S.); fightingwwei@163.com (W.Z.); liqing1128@njtech.edu.cn (Q.L.)

**Keywords:** hydrogels, frontal polymerization, chitosan, mechanical strength, degradation, micro-IR imaging

## Abstract

Chitosan (CS) is widely used in biomedical hydrogels due to their similarity to extracellular matrix. However, the preparation method of CS-based hydrogel suffers the drawbacks of tedious operation, time-consuming and energy consumption. Thus, there is an urgent need to develop a rapid synthesis pathway towards hydrogels. In this work, we used a modified CS as a cross-linking agent and acrylic acid (AA) as monomer to prepare a hydrogel through frontal polymerization (FP), which facilitates a facile and rapid method achieved in several minutes. The occurrence of pure FP was confirmed via the frontal velocity and temperature profile measurement. In addition, the as-prepared hydrogel shows excellent mechanical strength up to 1.76 MPa, and the Young’s modulus (ranging from 0.16 to 0.56 MPa) is comparable to human skin. The degradation mechanism is revealed by the micro-IR images through the distribution of the functional groups, which is attributed to the breakage of the ether bond. Moreover, the hydrogel exhibits excellent degradability, biocompatibility and antibacterial properties, offering great potentials in tissue engineering. We believe this work not only offers a facile and rapid FP method to fabricate a robust degradable hydrogel, but also provides an effective pathway for the investigation of the degradation mechanism at the chemical bond analysis level.

## 1. Introduction

Hydrogels are characterized by both their solid and liquid property, and they possess high similarity to an extracellular matrix [1,2]. Thus, they have attracted enormous interest in various methods of exploration in biomedicine and tissue engineering [3]. As a cationic, hydrophilic linear polysaccharide found in nature [4], chitosan (CS) has been widely used for preparing biomedical hydrogels due to their good biocompatibility, antibacterial properties and degradability [5,6,7]. Usually, glutaraldehyde cross-linking is used for the synthesis of CS-based hydrogels, but it suffers from the drawbacks of low mechanical strength and residual toxic substances [8].

Modification, copolymerization and grafting methods have been emerging to functionalize CS-based hydrogels. For instance, by grafting methoxy poly (ethylene glycol) on the CS derivatives, Wang et al. achieved hydrogels with both good pH sensitivity and bioavailability [9]. Although the hydrogel exhibits good properties, the grafting process is complicated. In another example, Ding et al. prepared self-healing and tough hydrogels by using the acrylamide-modified CS (AMCS) and oxidized alginate [10]. In this case, the AMCS favors the formation of Schiff base linkage, endowing the hydrogel with a self-healing property. The preparation underwent several freeze–thawing cycles. In addition, Cao et al. designed a macromolecule-crosslinked hydrogel via in situ polymerization of acrylamide and allyl glycidyl ether-functionalized CS, which serves as a macro-crosslinker [11]. The hydrogel was obtained after reaction for 24 h at 25 °C. Interestingly, with both the physical entanglements and topologically reconfigurable crosslinks in the hydrogel network, outstanding mechanical properties (elongation of 4610%, fracture strength of 326 kPa) was achieved. Obviously, modified CS can improve the mechanical property, as well as enable new functionality. However, the drawbacks of tedious operation, time-consuming and energy consumption still exist, which remain key challenges. Thus, there is an urgent needed to develop a rapid synthesis method towards robust CS-based hydrogels.

When it comes to a facile and rapid synthesis method, frontal polymerization (FP) stands out owing to its self-propagating polymerization mode [12]. FP is a self-sustained reaction where just an initial energy input is needed to initiate polymerization, then a propagated localized reaction zone promotes the conversion of monomers into polymers [13]. FP shares a series of advantages, such as simple operation without stirring, rapid reaction rate, energy-saving, and good uniformity of products [14]. Thus, FP has been widely explored both in the development of new FP modes and the synthesis of various polymers [15]. The former involves the latest microfluidics-based FP and 3D printing-based FP [13,16,17], while the latter focuses on a series of polymers, including gradient materials, nanocomposites, resins, functional gels, etc. [18]. Undoubtedly, FP offers an effective and energy-saving pathway for the synthesis of polymers [19].

**Scheme 1:**
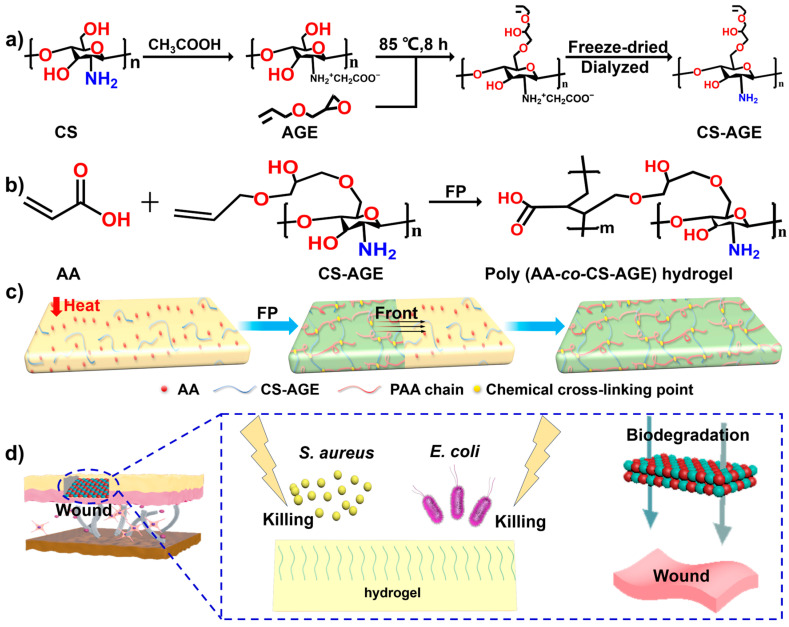
(**a**) Synthesis process of the CS-AGE. (**b**) Polymerization of AA and CS-AGE to prepare poly(AA-*co*-CS-AGE) hydrogels. (**c**) Schematic illustration for the synthesis of hydrogels via an FP process. (**d**) Hydrogels show promise for biocompatible, antimicrobial and biodegradable wound dressings.

In this work, we aim to obtain robust hydrogels in a facile and rapid FP manner. We synthesized allyl glycidyl ether (AGE)-modified CS (CS-AGE), which serves as a cross-linking agent (Scheme 1a). Meanwhile, acrylic acid (AA) was chosen as monomer due to a large number of carboxyl groups, which can increase the solubility of CS-AGE. Also, it can provide enough heat release to support the stable propagation of the polymerization. The poly (AA-co-CS-AGE) hydrogel was obtained via FP within several minutes (Scheme 1b,c). Interestingly, the hydrogel shows excellent mechanical strength up to 1.76 MPa, and Young’s modulus ranges from 0.16 to 0.56 MPa, which is equivalent to the Young’s modulus of human skin. In addition, we employed a micro-IR image measurement for the distribution analysis of chemical bond through the visual color variation, confirming that the breakage of the ether bond leads to degradation. The hydrogel also possesses excellent biocompatibility and antibacterial properties, offering great potential application in tissue engineering (Scheme 1d). This work offers the facile and rapid synthesis of robust degradable hydrogel by using a CS derivative through an FP technique, which holds great promise in the biological and medical field.

## 2. Results and Discussion

### 2.1. Fabrication of Poly(AA-co-CS-AGE) Hydrogels by FP

**Figure 1:**
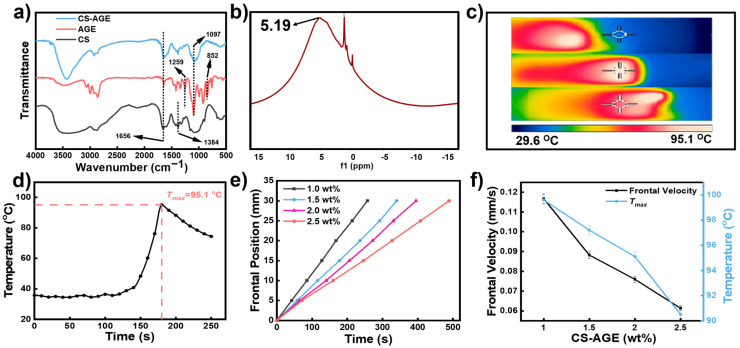
(**a**) FTIR spectra of CS, AGE and CS-AGE. (**b**) ^1^H NMR of CS-AGE. (**c**) IR images illustrate a stable front propagating upon time (left to right) during the synthesis of hydrogels via FP. (**d**) A typical temperature–time curve in FP process with CS-AGE mass ratio of 2 wt%. (**e**) Frontal position versus time in FP process under different CS-AGE concentrations. (**f**) Effect of CS-AGE mass ratio on the frontal velocity and *T_max_*.

In this work, we aim to fabricate antibacterial and degradable hydrogels via a rapid FP manner. To this end, we firstly modified CS with AGE, which renders the CS with an unsaturated double bond. Thus, the as-prepared CS-AGE can be copolymerized with other monomers by FP. The chemical structure of CS-AGE was characterized via Fourier transform infrared spectroscopy (FTIR) measurement. Figure 1a shows the FTIR spectra of CS, AGE and CS-AGE. The peaks at 1656 and 1384 cm^−1^ correspond to the vibrations of amide bond and methyl groups of CS, respectively. In the AGE spectrum, characteristic peaks at 1259 cm^−1^ and 852 cm^−1^ were observed, which belong to the epoxy vibrations of AGE. In particular, the disappearance of the characteristic epoxy peaks (1259 cm^−1^ and 852 cm^−1^) and the exist of characteristic peaks of CS (1656 and 1384 cm^−1^) confirm the successful modification of the CS with AGE. In addition, we used 1H-NMR spectra to characterize the chemical shifts of the hydrogen nuclei within CS-AGE (Figure 1b). There is a spectral line at a chemical shift of 5.19 ppm, corresponding to the chemical shift of the ethylene substrate (=CH2). The result confirms that AGE had undergone a ring cleavage reaction, and the double bond had been successfully introduced into the CS.

The second set of experiments focus on the exploration of poly(AA-*co*-CS-AGE) hydrogels via FP. Considering the reactivity and solubility of CS-AGE, here, AA was chosen as the monomer. AA possesses high reactivity, which can release a large amount of heat to propagate the polymerization [20]. In addition, there is a large number of carboxyl groups that could increase the solubility of CS-AGE (up to 3 wt%) [21]. We firstly investigated the pot life of the precursor mixture. It is found that it is inert at room temperature for more than 24 h. When heating one end with a soldering iron, a front occurs, which propagates through the whole system and promote the rapid conversion of the monomer to polymer. As shown in Figure 1c, the propagation of a stable front (from left to right) is observed using a thermal infrared radiation camera. From a typical temperature–time curve at a fixed position (CS-AGE, 2 wt%), a horizontal part, a maximum point and a decline line further confirm the occurrence of pure FP (Figure 1d). Typically, the horizontal part of the curve shows that the monomer does not react when the front does not propagate to the fixed position. The temperature reaches a maximum value (*T_max_*) of 95.1 °C when the front reaches the fixed position, while the temperature begins to decline when the front left due to thermal diffusion. Figure 1e shows the frontal position–time curves under different CS-AGE mass ratios. All the curves are straight lines demonstrating that the frontal velocity remains constant and that a pure FP reaction occurs. The results indicate that pure FP occurs without spontaneous polymerization. The effect of CS-AGE concentration on the frontal velocity and *T_max_* is shown in Figure 1f. When the CS-AGE concentration increases from 1.0 to 2.5 wt%, the frontal velocity decreases from 0.117 to 0.061 mm/s and the corresponding *T_max_* decrease from 99.6 to 90.5 °C.

### 2.2. Thermal Property and Mechanical Property of the Poly(AA-co-CS-AGE) Hydrogel

**Figure 2:**
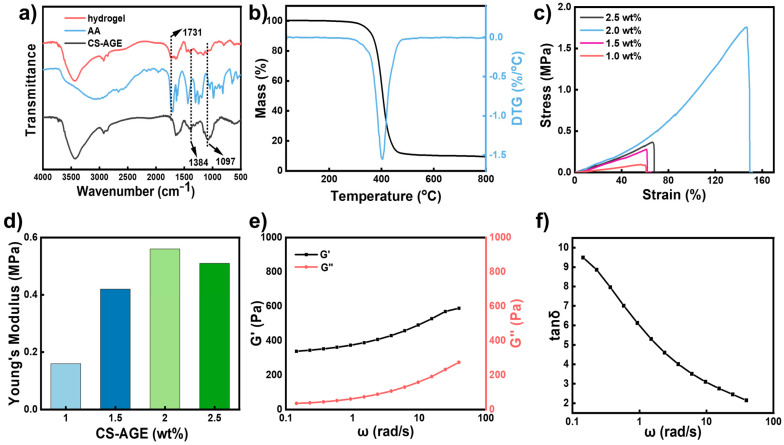
(**a**) FTIR spectra of CS-AGE, AA and poly(AA-*co*-CS-AGE) hydrogel. (**b**) TG and corresponding DTG curve of the hydrogel. (**c**) Stress–strain curves and (**d**) Young’s modulus of hydrogels with different CS-AGE mass ratios. (**e**) Storage modulus G′ and loss modulus G′′ of the hydrogels. (**f**) Loss tangent (tan δ) as a function of frequency of the hydrogel.

Figure 2a shows the FTIR spectrum of AA, CS-AGE and the as-prepared poly(AA-*co*-CS-AGE) hydrogel. The characteristic peak at 1731 cm^−1^ is assigned to the stretching vibration of carboxyl group of AA. The characteristic peak at 1384 cm^−1^ corresponds to the vibrations of methyl groups, while the peak at 1097 cm^−1^ is associated with the –C–O– or bridge-O stretch vibration of CS-AGE. All these characteristic peaks are observed in the spectra of the poly(AA-*co*-CS-AGE) hydrogel, indicating the cross-linking of AA and CS-AGE.

Figure 2b shows the TG curve and corresponding DTG curve of the hydrogel. Only one degradation process are found, which further indicates the successful synthesis of the poly(AA-*co*-CS-AGE) hydrogel. The degradation mainly occurs in the temperature range of 300~460 °C, corresponding to the backbone breakage, the decarboxylation process and complete breakdown of the polymer chain [22]. The mechanical properties of the prepared hydrogels were investigated by tensile tests. The stress–strain curves of the hydrogels prepared with different CS-AGE concentrations are shown in Figure 2c. The tensile strength and elongation at the break are augmented with an increasing amount of CS-AGE with maxima at 2 wt%, while a further increase gives a declining trend. In particular, when the CS-AGE mass ratio is 2 wt%, a maximum tensile strength of 1.76 MPa and elongation of 146% is obtained. The corresponding Young’s modulus of hydrogels ranges from 0.16 to 0.56 MPa (Figure 2d), which is equivalent to the Young’s modulus of human skin (0.42 to 0.85 MPa) [23]. We also measured the rheology of the hydrogel (Figure 2e). Typically, both the energy storage modulus G’ and loss modulus G” gradually increase with the frequency. Meanwhile, the G’ is larger than G” in the entire frequency range, indicating that the hydrogel network is completely elastic with a perfect covalent network [24]. In addition, the higher loss tangent (tan δ) is greater than 1 (Figure 2f), which demonstrates that the hydrogel has a high viscosity contribution. Overall, the as-synthesized poly(AA-*co*-CS-AGE) hydrogel exhibits excellent mechanical strength and a Young’s modulus comparable with human skin, which is promising in the field of tissue engineering.

### 2.3. Swelling Property and Microstructure of the Poly(AA-co-CS-AGE) Hydrogel

**Figure 3:**
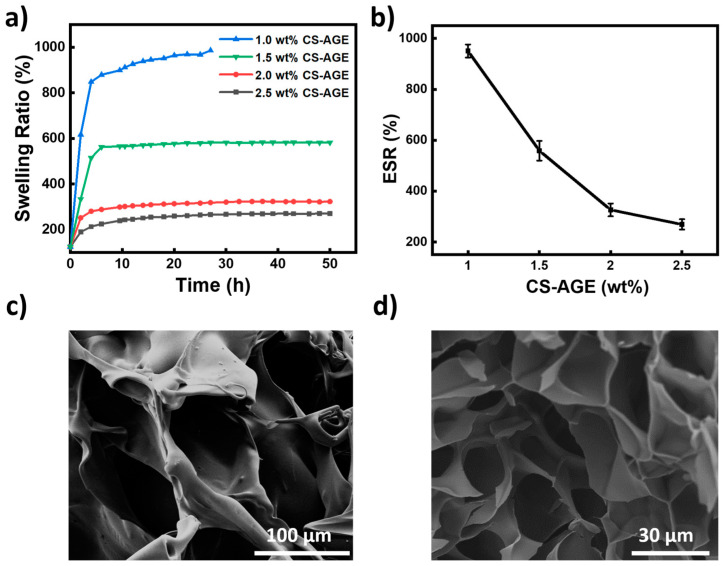
(**a**) Swelling ratio versus time and (**b**) ESR of the hydrogel with different CS-AGE mass ratios. SEM images of hydrogel sample with CS-AGE mass ratio of (**c**) 2 wt% and (**d**) 2.5 wt%.

Figure 3a shows the swelling properties of hydrogel samples prepared with different CS-AGE mass ratios in water. The swelling ratios increase with time and finally reach an equilibrium value, namely, the equilibrium swelling ratio (ESR). It is notably that the mass ratios of the CS-AGE have a significant impact on the ESRs. When the CS-AGE mass ratio is 1.0, 1.5, 2.0 and 2.5 wt%, the ESR is 985, 581, 323 and 271%, respectively (Figure 3b). The decrease Iin the ESR could be attributed to the high crosslink density of the hydrogel network [25]. To investigate the morphology of the hydrogels, the samples were analyzed by SEM (Figure 3c,d). For hydrogel with CS-AGE of 2 wt%, the sample shows a markedly porous structure with average pore size of 100 μm, which facilitates the migration and proliferation of cells such as dermal fibroblasts [26,27], whereas the hydrogel with CS-AGE of 2.5 wt% exhibits a porous structure with smaller pore size. The result is in consistent with the swelling properties that higher ESR corresponds to large pore size. Thus, it is predictable that the pore size of hydrogel becomes smaller with the increase in the CS-AGE mass ratio, which is ascribed to the higher crosslink density.

### 2.4. Degradability of the Poly(AA-co-CS-AGE) Hydrogel

**Figure 4:**
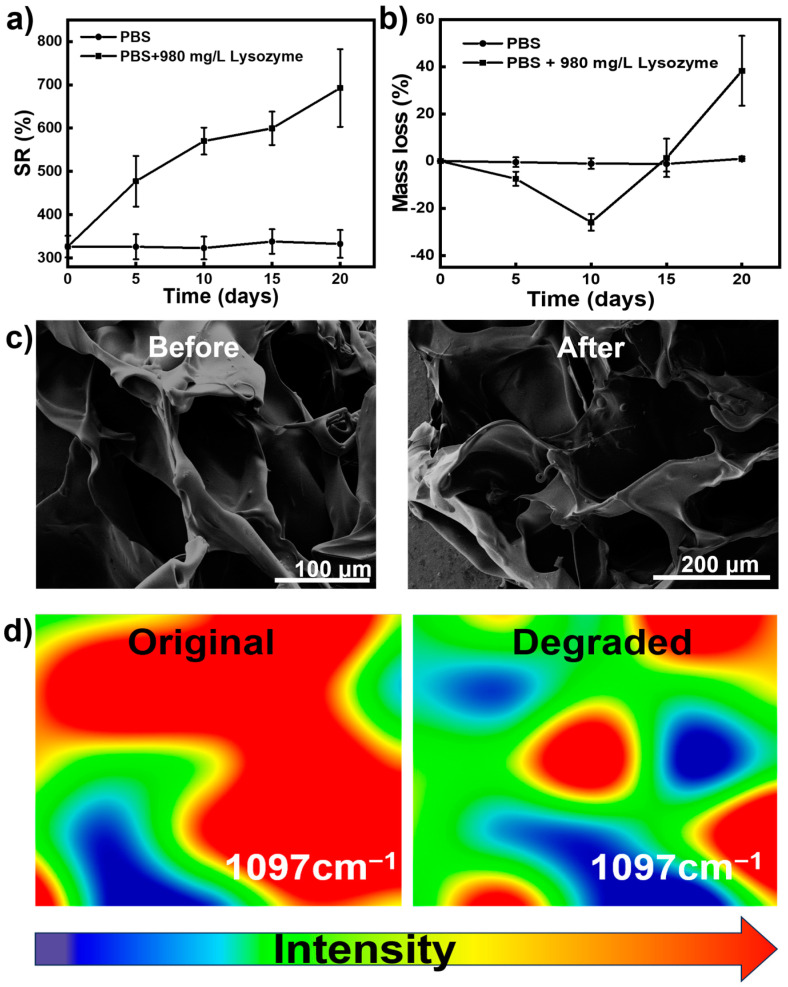
(**a**) SR and (**b**) mass loss as a function of time for in vitro degradation. (**c**) SEM images of hydrogels with CS-AGE mass ratio of 2 wt% before and after adding lysozyme. (**d**) Micro-IR images showing the ether bond (1097 cm^−1^) distribution density before and after degradation.

In vivo, CS is mainly depolymerized by lysozyme [28]. To simulate the degradation micro-environment in vivo, the hydrogel samples (CS-AGE = 2 wt%) were immersed in PBS solution with the addition of lysozyme. The ESR and mass loss over different times were measured, as shown in Figure 4a,b. It is clearly seen that the SR of the sample in the PBS solution is in consistent with swelling results as described above, while in the lysozyme-PBS solution, the SR gradually increases, which is up to 692.55% at 20 days (Figure 4a). It can be attributed to the continuous degradation with time, resulting in rearrangement of the network and a looser network, which is favorable to water absorption capacity [29]. From Figure 4b, it is found that the weight of the sample in PBS remains unchanged, indicating there is no degradability, whereas the weight of the sample in the lysozyme-PBS solution firstly increases and then declines after 10 days. Initially, the partial degradation with a small amount of glycosidic bond breaking occurred, and lysozyme attached at the recognition site, leading to a weight augment. With the degradation continuing, the polymer network became a loose flocculent structure. The generated small molecules were dissolved in the solution, and thus, the weight of the sample declined. We further analyzed the SEM images of the hydrogel with a CS-AGE mass ratio of 2 wt% before and after lysozyme treatment. As expected, the microscopic pore size is over 200 μm after lysozyme treatment, which is much larger than that of untreated sample (100 μm) (Figure 4c). The result further confirms that the poly(AA-*co*-CS-AGE) hydrogel degrades in the lysozyme-PBS solution [30]. For the purpose of the degradation mechanism, we collected the micro-IR images of the original and degraded sample. The micro-IR images offer a visual distribution of the functional groups, where the red color represents strong intensity, whereas the blue color reflects weak intensity. It is found that the original sample shows stronger red color at 1097 cm^−1^, corresponding to the ether bond, while for the degraded sample, the color becomes green and blue, indicating the weaker distribution of the ether bond (Figure 4d). The results illustrate that the degradation mechanism is based on the breakage of the ether bond. The micro-IR image measurement gives a new insight into the analysis of functional groups distribution, which provides an effective pathway for the investigation of degradation mechanism.

### 2.5. Biocompatibility and Antibacterial Property of the Poly(AA-co-CS-AGE) Hydrogel

**Figure 5:**
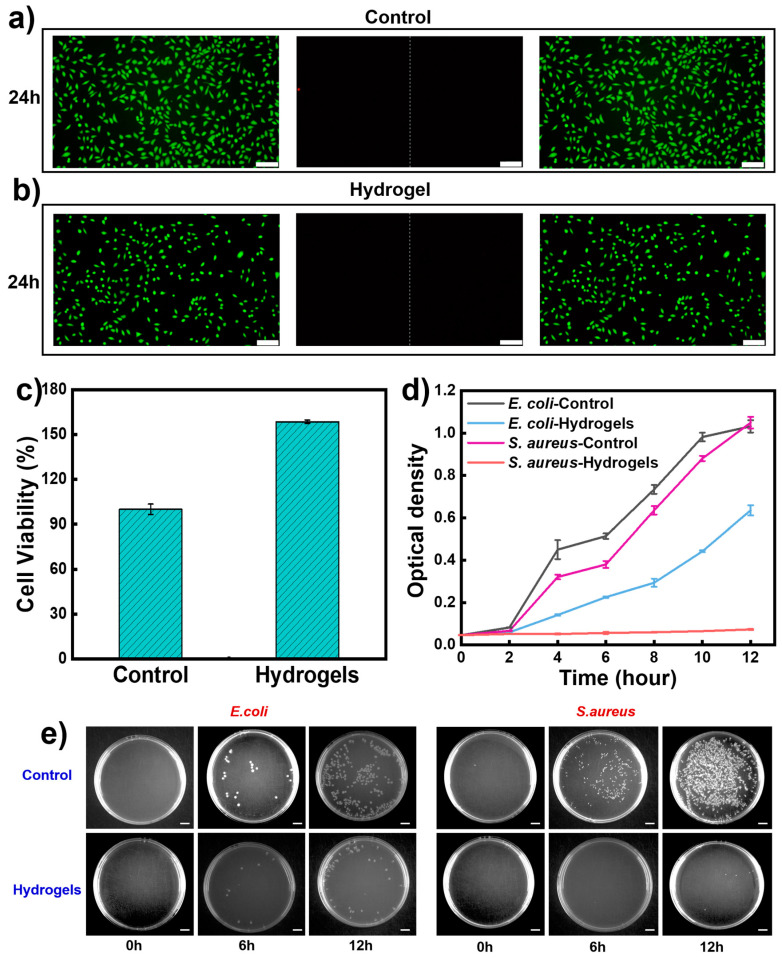
Live/dead staining of L929 fibroblasts after co-culture with (**a**) control and (**b**) hydrogel for 24 h, measured through a fluorescence microscope. Scale bar: 100 μm. (**c**) Cytocompatibility evaluation of hydrogels using the MTT assay. (**d**) Optical density measurements of bacteria *S. aureus* and *E. coli*. (**e**) Photographs demonstrating the bacterial inhibitory effect of the hydrogels against *S. aureus* and *E. coli*.

The biocompatibility of the hydrogel was tested via in vitro culture of L929 fibroblasts [31]. Live/dead staining was obtained via co-culture of L929 fibroblasts with control sample and hydrogel for 24 h, by employing a fluorescence microscope, to indicate the viability status of cells (Figure 5a,b). The proportion of dead cells is very low (red area) and most cells are alive and morphologically normal (green area). To provide a more precise insight into the collective state of the cells and obtain a quantitative measurement of the proliferation rate, the MTT assay was further carried out. As shown in Figure 5c, the survival rate of cells co-cultured with the hydrogel is significantly higher than that of the control group, which indicates that the hydrogel has good biocompatibility and low cytotoxicity.

Moreover, we investigated the antibacterial ability of the hydrogel by the OD value measurement, which refers to the absorption value of a solution at a wavelength of 600 nm. The OD value is proportional to the concentration of the absorbent substance in the solution. And the size and the shape of the bacteria/substance also plays an important role in the OD value [32]. Here, the Gram-negative (*E. coli*, rod-shaped, diameter of 0.5 μm, length of 1~3 0.5 μm) and Gram-positive (*S. aureus*, spherical, diameter of 0.8 μm) bacteria were chosen to be co-cultured with the hydrogel for 12 h. As shown in Figure 5d, it is clear that the hydrogel group displays a significant reduction in the number of bacteria compared to the control group. Inhibition of *E. coli* reaches 38.4%, while that of *S. aureus* reaches 92.3%. In addition, the coated plate method (Figure 5e) was further used to measure the bacterial inhibitory effect of the hydrogels. Visually, the hydrogel shows outstanding inhibition effect on bacteria, especially for the *S. aureus*. It should be noted that under the similar OD value, the concentration of the *E. coli* and the *S. aureus* is quite different, which could be attributed to the size and shape difference. These results indicate that the hydrogel could effectively inhibit the proliferation of *S. aureus*, while the inhibition of *E. coli* proliferation is weaker, which is consistent with the reported finding that the bactericidal effect of CS on Gram-positive bacteria is generally stronger than that of Gram-negative bacteria [33]. There are plenty of amino groups in CS, which can interact with bacterial cell walls, thus leading to the destruction of the bacterial cell walls and the death of the colonies. Also, it can inhibit the synthesis of DNA and RNA of bacteria, interfering with growth of bacteria [34]. All these results indicate that the hydrogel not only has excellent antibacterial capacity, but also has a positive effect on the proliferation of L929 cells, offering promise for tissue scaffolding material [33,35].

## 3. Conclusions

In summary, we developed a robust degradable poly(AA-*co*-CS-AGE) hydrogel through a facile and rapid manner via FP, where CS was modified through AGE to serve as a degradable cross-linking agent. The FP strategy facilitates the facile synthesis of the hydrogel within several minutes, which is time-saving and energy-efficient. The frontal velocity is consistent, and the temperature profile exhibits a maximum value. These findings confirm the occurrence of pure FP. The as-prepared hydrogel shows excellent mechanical strength up to 1.76 MPa, and the Young’s modulus ranges from 0.16 to 0.56 MPa, which is equivalent to the Young’s modulus of human skin. In addition, it possesses excellent degradability, biocompatibility and antibacterial properties, offering great potential application in tissue engineering. The degradation mechanism is illustrated by the micro-IR images, where the distribution of the ether bond (1097 cm^−1^) before and after degradation is measured. The distribution of the ether bond becomes weaker after degradation, indicating that the degradation is originated form the breakage of the ether bond. This work provides a rapid FP strategy for the fabrication of robust degradable hydrogels, which overcomes the drawbacks of complicated and tedious preparation process in other works. Meanwhile, the micro-IR image measurement might also provide an effective pathway for the investigation of the degradation mechanism at the chemical bond analysis level.

## 4. Materials and Methods

### 4.1. Materials

Acrylic acid (AA), Chitosan (CS, Mn = 1526.45, ~75% degree of deacetylation), allyl glycidyl ether (AGE), 2,2′-Azobis (2-methylpropionamidine) dihydrochloride (AAPH) and phosphate-buffered saline (PBS) were purchased from Aladdin Reagents Co., Ltd. in Shanghai, China. Glycerin and deionized water were purchased from Sinopharm Chemical Reagent Co., Ltd. in Shanghai, China. Deuterium oxide (D_2_O) was purchased from Energy Chemical in Shanghai, China. Lysozyme (40,000 U/mg, from egg white) was purchased from RHAWN in Shanghai, China. All reagents were used directly as received.

### 4.2. Preparation of CS-AGE

CS-AGE was synthesized and modified according to the literature [11]. Briefly, 3 g CS was added to aqueous acetic acid solution (150 mL, 2 wt%) and stirred continuously and vigorously to obtain a homogeneous solution. Then, the solution was transferred to a 250 mL three-necked flask, followed by adding 6 g AGE, stirring at high speed and heating to 85 °C for 8 h. The product was cooled to give a clear yellow viscous solution. Finally, the product was dialyzed for 1 week using a dialysis bag retaining a molar weight of 1000 Da, and the purified product was freeze-dried and stored under refrigeration.

### 4.3. Preparation of Poly(AA-co-CS-AGE) Hydrogels via FP

A certain amount of AA was mixed with glycerol and stirred evenly. CS-AGE in various mass ratios was then added to the solution, stirred at high speed, and heated to 70 °C until a homogeneous jelly-like fluid was obtained. After the solution was cooled, a small amount of AAPH was added and stirred until completely dissolved. A typical composition was AA = 60 wt%, CS-AGE = 2 wt%, glycerin = 38 wt%, AAPH = 0.02 wt% (the AAPH content is negligible for the total mass). The resulting homogeneous mixture was transferred to a rectangular plate template (100 mm × 25 mm × 7 mm) and one end of the plate was then heated with a soldering iron at 140 °C. Within 30 s there was a visible interface between the monomer and the polymer. The heat source was removed once the front had formed. Then, the front was propagating through the whole plate template. Finally, the resulting hydrogel was removed from the template for further study. Notably, when the CS-AGE content changes, the glycerin content remains unchanged at 38 wt%, and the total mass of CS-AGE and AA remains unchanged at 62 wt%.

### 4.4. Frontal Velocity and Temperature Measurement

The frontal position and corresponding propagating time were recorded to obtain a frontal position–time curve. Frontal velocity can be calculated by the slope. To obtain the temperature profile, a FLIR-E6390 thermal imager was used to measure the temperature at the fixed position.

### 4.5. Characterization

FTIR spectra and proton nuclear magnetic resonance spectroscopy (1H-NMR) were used to analyze the chemical structures of CS-AGE.

FTIR spectra were recorded using a Nicolet-6700 spectrometer at room temperature with Thermo Electron for 32 scans with 4 cm^−1^ resolution scans. (The hydrogel was immersed in deionized water for one week to remove unreacted monomers, and dried in an oven for 24 h.)

Scanning electron microscopy (SEM) images were obtained with a QUANTA 200 (Philips-FEI, Amsterdam, Holland) at 20.0 kV. (The hydrogel was immersed in deionized water for one week to reach an equilibrated swelling ratio, and freeze-dried for 24 h.)

The TGA test was measured using a TGA-DSC synchronous thermal analyzer with a heating rate of 1 °C/min and a heating range of 40–700 °C to obtain a thermogravimetric (TG) curve. The differential thermogravimetric (DTG) curve, the rate of mass change as a function of temperature, is obtained by recording the first derivative of the TG curve with respect to temperature. (The hydrogel was immersed in deionized water for one week and then dried in oven for 24 h.)

The swelling ratio of the hydrogel in water was obtained via weight analysis. Weighed hydrogel pieces were immersed in deionized water. After a certain interval, the samples were taken out, wiped with absorbent paper, weighed and then placed in the deionized water again. This procedure was repeated until the weight of the hydrogel remained essentially the same. The swelling ratio (SR) was calculated using the following equation:

$$SR=\frac{W_{t}-W_{0}}{W_{0}}\times 100\%$$

W_t_ is the weight of the hydrogel after water absorption, and W_0_ is the initial weight of the hydrogel.

The mechanical properties of the hydrogel were characterized by tensile tests with a SANS CMT6203 testing machine. Stress–strain curves were obtained at a crosshead speed of 10 mm/min at room temperature. The rheological measurements of hydrogels were tested on a modular compact rheometer (MCR302, Anton Paar, Austria). The specimens were made into discs with a thickness of 2 mm and a diameter of 2 cm. The test was carried out in the frequency range 0.1 to 50 Hz, and the deformation amplitude was set to γ_0_ = 0.1 to ensure that the oscillatory deformation was within the linear range. Each test was run at least three times to ensure feasibility.

In vitro degradation experiments of hydrogels were carried out under simulated physiological conditions. Briefly, lyophilized hydrogels were cut into small pieces and weighed. The hydrogel pieces were immersed in PBS (pH = 7.4) containing 980 mg/L lysozyme and maintained at an ambient temperature of 37 °C [36]. The soaking solution was changed daily to ensure enzyme activity. At determined time points (5, 10, 15 or 20 days), the samples were taken out, wiped with absorbent paper to remove excess water from the surface and weighed. The samples were then freeze-dried and weighed again. Mass loss was calculated using the following Equation:

Mass loss=×100%

W_de_ is the weight of the dried hydrogel after the simulated degradation treatment and W_0_ is the initial weight of the hydrogel in the dry state.

A Thermo Scientific Nicolet iN10 infrared microscope was employed to record micro-IR images of hydrogels under reflection mode equipped with a liquid nitrogen-cooled detector.

The biocompatibility of the hydrogels was examined using live/dead staining and MTT methods. Live/dead staining was conducted on L929 cell cultures following exposure to hydrogel extracts. Specifically, L929 cells were seeded at a density of 5000 cells per well in a 96-well plate and allowed to adhere overnight. Subsequently, the cells were treated with hydrogel extracts for a duration of 24 h. To evaluate cell viability, the Calcein/PI Cell Viability/Cytotoxicity Assay Kit for mammalian cells (Beyotime, Shanghai, China) was utilized. After staining according to the manufacturer’s instructions, the cell samples were imaged using a fluorescence microscope. Alternatively, in the MTT assay, L929 cell cultures with hydrogel extracts were performed following the similar procedure mentioned above, and then 20 μL of MTT solution (5 mg/mL) was added to each well and the incubation was continued for 4 h. Afterward, 200 μL of Formazan solution was added to each well, thoroughly mixed, and further incubated for 4 h at 37 °C within the cell culture incubator. The absorbance was measured at 570 nm using a microplate reader (BioTek Synergy H1 Multimode Reader), and cell viability was calculated as a percentage relative to the control group.

The bacterial OD value assay was used to determine the antibacterial ability of the hydrogel against *E. coli* and *S. aureus*. 0.1 g of the treated hydrogel (immersing in PBS solution for 4 h and sterilizing via UV irradiation) was incubated with the bacterial solution and incubated for 4 h at 150 rpm and 35 °C with constant-temperature shaking. The control group (CG) was added with the same mass of deionized water. The reaction mixture was then transferred to 15 mL tubes containing 10 mL of LB medium per tube and then incubated in a shaking incubator at 150 rpm and 35 °C. The OD values at 600 nm wavelength were measured on a UV-visible spectrophotometer (Novaspec Plus). Bacterial regeneration was investigated by plotting the OD values against time, and bacterial growth dynamics were investigated. All experiments were repeated three times and the average values were recorded. In addition, the suspension was taken out with the dilution end point at 10^6^ and seeded on a nutrient agar plate to calculate the bacterial colonies after a certain time.

## Data Availability

All data and materials are available on request from the corresponding author. The data are not publicly available due to ongoing research using a part of the data.
